# Supplementary material for: DMETTM Genotyping: Tools for Biomarkers Discovery in the Era of Precision Medicine
Source: High Throughput. 2020 Mar 29;9(2):8. doi: 10.3390/ht9020008 (PMC7362183; doi:10.3390/ht9020008)
Supplement: Supplementary file 1 [file high-throughput-09-00008-s001.pdf]

Table S1. List of genes included in the DMET Plus panel.

ABCB1	*ALDH2*	*COMT*	*CYP4F3*	*EPHX2*	*MAOA*	*RPL13*	*SLC22A14*	*TBXAS1*
ABCB4	*ALDH3A1*	*CROT*	*CYP4F8*	*FAAH*	*MAOB*	*RXRA*	*SLC25A27*	*TPMT*
ABCB7	*ALDH3A2*	*CYP1A1*	*CYP4F11*	*FMO1*	*MAT1A*	*SERPINA7*	*SLC28A1*	*TPSG1*
ABCB11	*AOX1*	*CYP1A2*	*CYP4F12*	*FMO2*	*METTL1*	*SLC5A6*	*SLC28A2*	*TYMS*
ABCC1	*APOA2*	*CYP1B1*	*CYP4Z1*	*FMO3*	*NAT1*	*SLC6A6*	*SLC28A3*	*UGT1A1*
ABCC2	*ARNT*	*CYP2A6*	*CYP7A1*	*FMO4*	*NAT2*	*SLC7A5*	*SLC29A1*	*UGT1A3*
ABCC3	*ARSA*	*CYP2A7*	*CYP7B1*	*FMO5*	*NNMT*	*SLC7A7*	*SLC29A2*	*UGT1A4*
ABCC4	*ATP7A*	*CYP2A13*	*CYP8B1*	*FMO6*	*NQO1*	*SLC7A8*	*SLCO1A2*	*UGT1A5*
ABCC5	*ATP7B*	*CYP2B6*	*CYP11A1*	*G6PD*	*NR1I2*	*SLC10A1*	*SLCO1B1*	*UGT1A6*
ABCC6	*CA5P*	*CYP2B7P1*	*CYP11B1*	*GSTA1*	*NR1I3*	*SLC10A2*	*SLCO1B3*	*UGT1A7*
ABCC8	*CBR1*	*CYP2C8*	*CYP11B2*	*GSTA2*	*NR3C1*	*SLC13A1*	*SLCO2B1*	*UGT1A8*
ABCC9	*CBR3*	*CYP2C9*	*CYP17A1*	*GSTA3*	*ORM1*	*SLC15A1*	*SLCO3A1*	*UGT1A9*
ABCG1	*CDA*	*CYP2C18*	*CYP19A1*	*GSTA4*	*ORM2*	*SLC15A2*	*SLCO4A1*	*UGT1A10*
ABCG2	*CES2*	*CYP2C19*	*CYP20A1*	*GSTA5*	*PGAP3*	*SLC16A1*	*SLCO5A1*	*UGT2A1*
ABP1	*CHST1*	*CYP2D6*	*CYP21A2*	*GSTM1*	*PNMT*	*SLC19A1*	*SPG7*	*UGT2B4*
ADH1A	*CHST2*	*CYP2E1*	*CYP24A1*	*GSTM2*	*PON1*	*SLC22A1*	*SPN*	*UGT2B7*
ADH1B	*CHST3*	*CYP2F1*	*CYP26A1*	*GSTM3*	*PON2*	*SLC22A2*	*SULT1A1*	*UGT2B11*
ADH1C	*CHST4*	*CYP2J2*	*CYP26C1*	*GSTM4*	*PON3*	*SLC22A3*	*SULT1A2*	*UGT2B15*
ADH4	*CHST5*	*CYP2S1*	*CYP27A1*	*GSTM5*	*POR*	*SLC22A4*	*SULT1A3*	*UGT2B17*
ADH5	*CHST6*	*CYP3A4*	*CYP27B1*	*GSTO1*	*PPARD*	*SLC22A5*	*SULT1B1*	*UGT2B28*
ADH6	*CHST7*	*CYP3A5*	*CYP39A1*	*GSTP1*	*PPARG*	*SLC22A6*	*SULT1C2*	*UGT8*
ADH7	*CHST8*	*CYP3A7*	*CYP46A1*	*GSTT1*	*PPP1R9A*	*SLC22A7*	*SULT1C4*	*VKORC1*
AHR	*CHST9*	*CYP3A43*	*CYP51A1*	*GSTT2*	*PRSS53*	*SLC22A8*	*SULT1E1*	*XDH*
AKAP9	*CHST10*	*CYP4A11*	*DCK*	*GSTZ1*	*PTGIS*	*SLC22A11*	*SULT2A1*	
ALB	*CHST11*	*CYP4B1*	*DPYD*	*HMGCR*	*QPRT*	*SLC22A12*	*SULT2B1*	
ALDH1A1	*CHST13*	*CYP4F2*	*EPHX1*	*HNMT*	*RALBP1*	*SLC22A13*	*SULT4A1*	
